# The Influence of Physical Properties on the Membrane Morphology Formation during the Nonisothermal Thermally Induced Phase Separation Process

**DOI:** 10.3390/polym15163475

**Published:** 2023-08-19

**Authors:** Samira Ranjbarrad, Philip K. Chan

**Affiliations:** Department of Chemical Engineering, Toronto Metropolitan University, 350 Victoria Street, Toronto, ON M5B 2K3, Canada; samira.ranjbar@torontomu.ca

**Keywords:** thermally induced phase separation, membrane, spinodal decomposition, Cahn–Hilliard equation, polymers

## Abstract

The physical properties of a polymer solution that are composition- and/or temperature-dependent are among the most influential parameters to impact the dynamics and thermodynamics of the phase separation process and, as a result, the morphology formation. In this study, the impact of composition- and temperature-dependent density, heat capacity, and heat conductivity on the membrane structure formation during the thermally induced phase separation process of a high-viscosity polymer solution was investigated via coupling the Cahn–Hilliard equation for phase separation with the Fourier heat transfer equation. The variations of each physical property were also investigated in terms of different boundary conditions and initial solvent volume fractions. It was determined that the physical properties of the polymer solution have a noteworthy impact on the membrane morphology in terms of shorter phase separation time and droplet size. In addition, the influence of enthalpy of demixing in this case is critical because each physical property showed a nonhomogeneous pattern owing to the heat generation during phase separation, which in turn influenced the membrane morphology. Accordingly, it was determined that investigating spinodal decomposition without including heat transfer and the impact of physical properties on the morphology formation would lead to an inadequate understanding of the process, specifically in high-viscosity polymer solutions.

## 1. Introduction

The characteristics of the porous polymeric membranes, such as pore shape and size, strongly depend on the operating parameters, system properties, kinetics, dynamics, and thermodynamics of the phase separation process [1,2,3,4,5,6]. When investigating spinodal decomposition, it is important to consider the effect of heat transfer on the phase separation process due to the nonisothermal nature of the process [7,8,9,10,11,12]. Heat transfer can have a significant impact on the cooling rate and, therefore, on the morphology formation [7,8,9,10,11,12,13,14,15,16,17]. Additionally, the physical properties of the polymeric system, such as viscosity, density, heat conductivity, heat capacity, etc., can also impact the morphology formation in terms of the variations in temperature and concentration during phase separation [18,19,20,21,22]. In order to gain a broad understanding of the phase separation mechanism, it is important to consider the influence of each physical property on the formation of the morphological domains in membranes [20,21,22]. Failure to consider the effect of heat transfer and the role of physical parameters would lead to an incomplete understanding of the process and, as a result, inaccurate predictions or conclusions about the behavior of polymeric membranes. In addition, understanding the interaction between the physical properties and their dependence on concentration and/or temperature will elucidate and control the phase separation process more precisely to produce membranes with specific characteristics, e.g., void volume, pore shape, isotropy or anisotropy, etc. [18,19,20,21].

Each physical property of a polymer solution can have a direct influence on heat transfer and, accordingly, on phase separation of the polymeric system. For example, the concentration of the polymer in the solution can also lead to a slower heat transfer rate by increasing the viscosity [23]. Likewise, higher thermal conductivity can result in a faster rate of heat transfer [20]. The difference in heat conductivities between polymer and solvent can influence phase separation and morphology formation by affecting the cooling rate. This difference in cooling rates can entail a temperature gradient within the system, which can influence the thermodynamics of the system and the kinetics of phase separation. Overall, the combination of all these properties makes the polymeric system complex to predict and model, which emphasizes the significance of considering all the aspects involved during phase separation [20].

In our previous studies, we coupled the nonlinear Cahn–Hilliard equation with the Fourier heat transfer equation and a transient heat equation with the aim of investigating the impact of heat transfer on phase separation during the thermally-induced phase separation process in terms of applying various boundary conditions and initial solvent volume fractions. It was observed that heat transfer impacted the phase separation dramatically, and it was concluded that the effect of heat transfer and the heat of demixing could not be ignored during phase separation [8,9].

In this study, the influence of the temperature- and composition-dependent physical properties of the system, namely density, heat capacity, and heat conductivity, on the membrane structure formation is investigated during the TIPS process and compared with the results of our previous studies, where the physical properties were kept constant during phase separation. By incorporating all the concentration- and temperature-dependent parameters into the model development, we anticipate that the adjustments made to our previous model will yield an improved representation of the intricacies involved in phase separation dynamics and thermodynamics.

The impact of the physical properties of a system on phase separation through spinodal decomposition has not been widely investigated in the literature [13,20,21,22]. The impact of a composition-dependent gradient energy parameter was investigated by Molin and Mauri [13] for a high-viscosity polymer solution. They observed that the difference in the heat conductivities of the solvent and the polymer led to an anisotropic structure, which was influenced by heat and mass transfer rates inside the system. Atkinson and Lloyd [24] investigated membrane formation through the thermally induced phase separation process theoretically and experimentally, while considering the temperature-dependent properties of the system in their model. They simulated the evaporation stage to produce anisotropic morphology before phase separation initiation. Matsuyama et al. [14] studied the impacts of a quenching medium, the quench temperature, and the cooling rate on the formation of anisotropic membranes using the thermally induced phase separation process. They found that evaporating the solvent from one side while immersing the other side in ice water would create phase separation. As a result, the upper part of the membrane (which had been partially evaporated) had smaller pores, and the lower part of the membrane (which had a lower polymer concentration) had larger pores. Huston et al. [17] included the temperature-dependent coefficients in the Cahn–Hilliard equation for continuous cooling through the phase separation process. Ariyapadi et al. [25] derived the entropic contributions of the gradient energy parameter for binary and ternary polymer systems. Miranville et al. [26] proposed a nonisothermal Cahn–Hilliard equation for phase separation that included the temperature-dependent properties in the Cahn–Hilliard equation.

In the TIPS process, a polymer and a low-molecular-weight, high-boiling-point solvent are combined at an elevated temperature to create a homogeneous mixture [1,2,3,4,5]. A sudden decrease in temperature in a system with an upper critical solution temperature (UCST) causes phase separation through a rapid cooling process, leading the system to enter the two-phase region of the phase diagram and separate into regions of high and low polymer concentrations. Finally, the medium is frozen, and the solvent is removed [4,5,6]. Depending on the e temperature and concentration, liquid–liquid phase separation can be achieved through either spinodal decomposition or nucleation and growth. Spinodal decomposition is a process that can be divided into three stages based on the time development of the sinusoidal wave patterns, which represent the one-dimensional concentration fluctuations of the system. These stages are referred to as the early, intermediate, and late stages [8,9,16,27]. During the early stage, the spatial concentration fluctuations have a fixed wavelength, but their amplitude increases over time. During the intermediate stage, both the wavelength and amplitude of the spatial concentration fluctuations increase as time progresses. While the wavelength increases over time, which results in coarser membrane pores, the amplitude in the late stage is at its equilibrium composition [15,16].

During this process, the temperature changes with time, and this time-dependent temperature evolution can impact the membrane structure formation, depending on the process conditions [28]. The early stages of phase separation can evolve very rapidly, and the kinetics of the process can be highly non-linear. This can make it challenging to accurately predict the behavior of the system [2,29]. To overcome these limitations, researchers use numerical simulations to study the system at smaller length and time scales, which can provide valuable information about the underlying physics of the system, and this information can be used to develop more efficient and accurate predictive models [30].

## 2. Model Development

In this section, the model is derived by combining the nonlinear Cahn–Hilliard equation for phase separation dynamics and the Fourier heat transfer equation in two dimensions, while taking into account the temperature- and composition-dependent variables in the Fourier heat transfer equation. Given that the current model builds upon the foundation established in our prior studies [8,9], we have opted to omit the elucidation of the equation derivations within this paper. Instead, these derivations are presented in a concise format within Table 1.

The mathematical model employed in this paper is nondimensionalized using the scaling relations below:

(8) $$c^{*}=c$$

(9) $$T^{*}=\frac{T}{\theta }$$

(10) $$x^{*}=\frac{x}{L}$$

(11) $$y^{*}=\frac{y}{L}$$

(12) $$t^{*}=\frac{k_{B}\theta R_{g}^{2}}{\zeta L^{4}}t$$

(13) $$l^{*}=\frac{l}{L}$$

(14) $$\Lambda =\frac{L^{2}}{{NR}_{g}^{2}}$$

(15) $$\alpha =\frac{\zeta L^{2}}{k_{B}\theta R_{g}^{2}}$$

where *L* is the length of the membrane in the dimension of the square *L* × *L* and t* is the dimensionless time.

**Figure 1:**
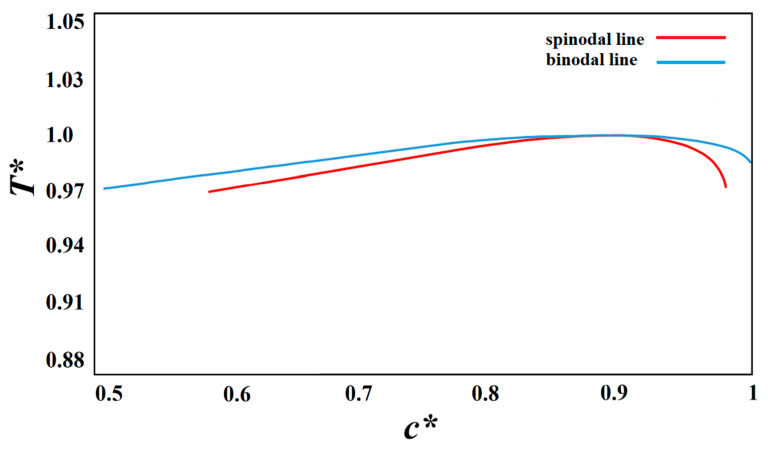
Schematic representation of the phase diagram for polystyrene–cyclohexanol solution with *N*_2_ = 100.

The solution used in this study is the polystyrene–cyclohexanol polymer solution. The phase diagram for this system with a degree of polymerization of *N*_2_ = 100 is plotted in Figure 1 using the Flory–Huggins free energy density adopted from Kurata [42], which is consistent with the experimental phase diagrams for the polystyrene–cyclohexanol polymer solution available in the literature [5,19,21,23,43,44,45]. Since the viscosity of the polymer is highly dependent on molecular weight and temperature, the exact value is not provided at the temperature range studied. However, the viscosity of polystyrene in cyclohexanol is equal to 56.45 cP at 25 °C [46].

The following Cahn–Hilliard equation is derived using the scaling relationships listed above and the composition- and temperature-dependent parameters:

$$\frac{\partial c^{*}}{\partial t^{*}}={\Lambda T}^{*}\left(\frac{1}{N_{2}}-1-2\chi \left(1-2c^{*}\right)\right)\left(\frac{\partial c^{*}}{\partial x^{*}}\frac{\partial c^{*}}{\partial x^{*}}+\frac{\partial c^{*}}{\partial y^{*}}\frac{\partial c^{*}}{\partial y^{*}}\right)$$

(16) $$+{\Lambda T}^{*}\left(\left(1-c^{*}+\frac{c^{*}}{N_{2}}-2\chi \left(1-2c^{*}\right)\right)\left(\frac{\partial^{2}c^{*}}{\partial {x^{*}}^{2}}+\frac{\partial^{2}c^{*}}{\partial {y^{*}}^{2}}\right)\right)-\frac{T^{*}\chi }{3}$$

$$\left(1-2c^{*}\right)\left\{\frac{\partial c^{*}}{\partial x^{*}}\left(\frac{\partial^{3}c^{*}}{\partial {x^{*}}^{3}}+\frac{\partial^{3}c^{*}}{\partial x^{*}\partial {y^{*}}^{2}}\right)+\frac{\partial c^{*}}{\partial y^{*}}\left(\frac{\partial^{3}c^{*}}{\partial {y^{*}}^{3}}+\frac{\partial^{3}c^{*}}{\partial y^{*}\partial {x^{*}}^{2}}\right)\right\}$$

$$-\frac{T^{*}\chi }{3}c^{*}(1-c^{*})(\frac{\partial^{4}c^{*}}{\partial {x^{*}}^{4}}+\frac{2\partial^{4}c^{*}}{\partial {x^{*}}^{2}\partial {y^{*}}^{2}}+\frac{\partial^{4}c^{*}}{\partial {y^{*}}^{4}})$$

Taking into account the infinitesimal thermal concentration fluctuations that exist in the homogeneous solution is the most appropriate initial condition for solving the Cahn–Hilliard equation. The dimensionless expression for the initial concentration is given as [1]:

(17) $$c^{*}\left(t^{*}=0\right)={c^{*}}_{0}+\delta c^{*}\left(t^{*}=0\right)$$

where ${c^{*}}_{0}$ is the dimensionless average concentration of the solvent and $\delta c^{*}(t^{*}=0)$ presents the initial thermal concentration fluctuations of the system at equilibrium, which is considered to be $\pm 10^{-6}$ in this study. Thermal fluctuations are minute changes in component concentration brought on by the random movement of molecules. They can be thought of as the origin of the formation of a phase separation process because they can be found even in a homogeneous solution.

The nonperiodic boundary conditions offer an accurate simulation of the phase separation process [1,8,9].

The zero-mass flux boundary condition, which is a type of non-periodic boundary condition, is an appropriate option since no mass is transmitted through the surfaces in systems with fixed boundaries, such as droplets or thin films. This boundary condition is used in this work to ensure that the mass of the component is conserved within the simulation domain. The following are the zero-mass flow boundary conditions applied in this study [1,15,27]:

(18) $$\frac{{\partial^{3}c}^{*}}{\partial {x^{*}}^{3}}+\frac{{\partial^{3}c}^{*}}{\partial {x^{*}}^{2}\partial {y^{*}}^{2}}=0,\;\mathrm{at}\;t^{*}>0\;\mathrm{and}\;x^{*}=0\;\mathrm{and}\;x^{*}=1$$

(19) $$\frac{{\partial^{3}c}^{*}}{\partial {y^{*}}^{3}}+\frac{{\partial^{3}c}^{*}}{\partial {y^{*}}^{2}\partial {x^{*}}^{2}}=0,\;\mathrm{at}\;t^{*}>0\;\mathrm{and}\;y^{*}=0\;\mathrm{and}\;y^{*}=1$$

A natural boundary condition resulting from the shift in free energy is chosen as the second set of boundary conditions. The natural boundary conditions are presented as follows [1,15,27]:

(20) $$\left[\nabla^{*}c^{*}\right]\cdot n=0$$

where **n** is the outward unit normal and is expressed in Cartesian coordinates as follows:

(21) $$\frac{{\partial c}^{*}}{\partial x^{*}}=0,\;\mathrm{at}\;t^{*}>0\;\mathrm{and}\;x^{*}=0\;\mathrm{and}\;x^{*}=1$$

(22) $$\frac{{\partial c}^{*}}{\partial y^{*}}=0,\;\mathrm{at}\;t^{*}>0\;\mathrm{and}\;y^{*}=0\;\mathrm{and}\;y^{*}=1$$

The following Fourier heat transfer equation is utilized to account for the temporal and spatial temperature gradients in the *x*- and *y*-directions [47]:

(23) $$(\rho_{s}c+\rho_{p}\left(1-c\right))\times (c_{p,s}c+c_{p,p}\left(1-c\right))\frac{\partial T^{*}}{\partial t^{*}}=(k_{s}c+k_{p}\left(1-c\right))\left(\frac{\partial^{2}T^{*}}{\partial {x^{*}}^{2}}+\frac{\partial^{2}T^{*}}{\partial {y^{*}}^{2}}\right)+\dot{q}$$

where the energy dissipation term, $\dot{q}$, is regarded as the enthalpy of demixing [13,20,48]. Subscript *s* denotes the solvent, and subscript *p* denotes polymer. Density ρ, heat capacity $c_{p}$ and heat conductivity k, are supposed to be concentration- and temperature-dependent. Table 2 summarizes the equations used to designate the temperature- and composition-dependence of each physical property.

For a system with an initial polymer composition of *c*_0_ and phase-separated compositions of *c*_1_ and *c*_2_, the lever rule and Flory–Huggins theory are used to determine the difference in the mixing enthalpy between the original system and the demixed system as follows [43,44,53,54]:

(24) $${∆H}_{demix}=\frac{k_{B}T\chi }{{\nu (c}_{2}-c_{1})}\left[\begin{array}{c} {(c}_{2}-c_{0})(1-c_{1})+{(c}_{0}-c_{1})c_{2}(1-c_{2})\\ -{(c}_{2}-c_{1})c_{0}(1-c_{0}) \end{array}\right]$$

The initial condition for the Fourier heat transfer equation represents the polymer solution that is used to fabricate the membrane initially at a high temperature and in a homogenous state before it is quenched into the unstable spinodal region. To study how morphology forms in response to different quench conditions, three sets of boundary conditions were used. Table 3 provides information on the boundary conditions and the initial compositions. The initial solvent volume fraction was defined as c*, the quench temperature was termed T*_q_, and the initial solution temperature was denominated as T*. T*_q__1_ and T*_q__2_ denote the two different quench temperatures that are applied to the two opposite sides of the membrane.

The dimensionless Fourier heat transfer equation, which includes temperature gradients in both the *x*- and *y*-directions, can be expressed in the following form:

$$\frac{\partial T^{*}}{\partial t^{*}}=\alpha \left[\frac{\left(1-0.94T\right)\left(1-c\right)+\left(0.17-0.07T\right)c}{\left[\frac{c}{0.26546^{{\left(1+\left(1-\frac{T}{2}\right)\right)}^{0.2848}}}+\left(12.9-2.14T-0.2T^{2}\right)\left(1-c\right)\right]}\right]\times$$

(25) $$\left[\frac{1}{\left[\left(2.2+1.65T\right)\left(1-c\right)+\left(-1+14.3T-12.65T^{2}+4.4T^{3}\right)c\right]}\right]\left(\frac{\partial^{2}T^{*}}{\partial {x^{*}}^{2}}+\frac{\partial^{2}T^{*}}{\partial {y^{*}}^{2}}\right)+$$

$$\frac{T^{*}\chi }{\left[\frac{c}{0.26546^{{\left(1+\left(1-\frac{T}{2}\right)\right)}^{0.2848})}}+\left(12.9-2.14T-0.2T^{2}\right)\left(1-c\right)\right]}\times$$

$$\left[\frac{1}{\left[\left(2.2+1.65T\right)\left(1-c\right)+\left(-1+14.3T-12.65T^{2}+4.4T^{3}\right)c\right]}\right]$$

$$\left[\begin{array}{c} {({1-c}^{*}}_{1}-{c^{*}}_{0})(1-{c^{*}}_{1})+{(c^{*}}_{0}-{c^{*}}_{1})(1-{c^{*}}_{1})(1-2{c^{*}}_{1})-\\ \left(1-2{c^{*}}_{1}){c^{*}}_{0}(1-{c^{*}}_{0}\right) \end{array}\right]\dot{c^{*}}$$

The membrane studied here is represented by a 2-D square geometry, which is discretized into an 80 × 80 mesh for the numerical simulation. The Galerkin finite element method incorporating Hermitian basis functions for spatial discretization was used to solve the governing equations. This resulted in two sets of time-dependent ordinary differential equations, which were solved using the Newton–Raphson method and integrated in time using the forward-Euler backward-Euler method [1,8]. The difference between the two successive solutions was chosen to be less than 10^−6^, according to the established convergence criterion. The process simulation was carried out using the Fortran programming language.

## 3. Results and Discussion

Simulation results for the three cases given in Table 3 are provided and discussed in this section. The direction of the temperature gradient, which is the rate of change of temperature in a given direction, is normal to the walls of the membrane.

**Figure 2:**
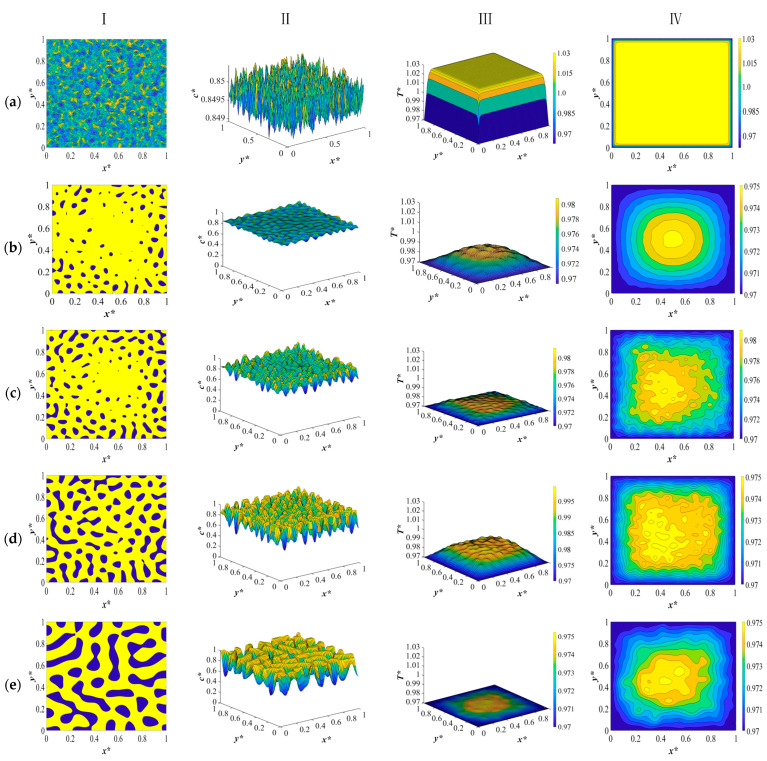
The dimensionless concentration (column I and II) and temperature profiles and patterns (column III and IV) for an off-critical quench with c* = 0.85 and T* = 1.03 and T*_q_ = 0.97 at the following times: (**a**) t* = 1.03 × 10^−6^, (**b**) t* = 3.03 × 10^−5^, (**c**) t* = 2.03 × 10*^−^*^4^, (**d**) t*= 8.03 × 10^−4^, and (**e**) t* = 8.03 × 10^−2^.

The dimensionless concentration and temperature profiles and patterns for an off-critical quench of the polymer solution with an initial solvent volume fraction of c* = 0.85 and the initial dimensionless solution temperature of T* = 1.03 quenched to the temperature of T*_q_ = 0.97 are provided in Figure 2. The initial concentration and initial and quench temperatures are selected from our previous studies so that the change in concentration and temperature due to a change in the physical properties of the system can be used to provide a comparison with regard to the influence of the physical properties on phase separation and the morphology formation [8,9]. In addition, due to the number of graphs in each section, only the results of the simulations for the model developed based on the composition- and temperature-dependent physical properties are provided in this paper to avoid misperception. In order to evaluate the structure formation during the phase separation in the first step, a quench was applied simultaneously from all four sides of the membrane.

As presented in Figure 2a, at time equal to t* = 1.03 × 10^−6^, the initial thermal concentration fluctuations still exist in the one phase region of the phase diagram, and the corresponding temperature profile represents a very short time after the quench is applied. The reason for providing the very early times is to provide the range of change in solution density, heat conductivity, and heat capacity in the initial solution at a high temperature and right after the quench is applied, which will be discussed in the following paragraphs.

All sides of the membrane are in contact with a cooling medium and are quenched to the same temperature, while the interior parts of the sample still retain the initial temperature due to the high viscosity of the solution [23]. Figure 2b represents the phase-separated profiles and patterns at time t* = 3.03 × 10^−5^. The droplets start to form at the sides where the quench is applied, while in the interior parts, no major phase separation can be detected. The yellow region represents the polymer-rich phase, and the blue region represents the solvent-rich droplets. The corresponding temperature profile in Figure 2b shows that there is a small amount of heat generated as a result of the enthalpy of demixing. Comparing these results with the dimensionless concentration and temperature patterns for the deep quench in our previous study reveals that phase separation has progressed more with smaller droplets being formed from the sides of the sample. In addition, the temperature profile exhibits more progress inside the sample, which also leads to more heat generation during this stage. This is due to the fact that considering the physical properties leads to an increase in heat diffusivity during phase separation, which is subject to the change in density, heat capacity, and thermal conductivity. Higher heat diffusivity means a higher heat transfer rate, which in turn leads to more phase separation. This phenomenon will be further elaborated.

At t* = 2.03 × 10^−4^, as shown in Figure 2c, phase separation has progressed in the interior parts of the membrane, with droplets being formed in the entire sample. The droplets that are formed are smaller in size compared to our previous study, where a constant heat diffusivity was used. In addition, the difference in the size of droplets between the interior parts and the boundaries is more vivid. The inhomogeneity in the corresponding temperature profile accounts for the heat that is released during phase separation as a result of demixing. The amount of heat generation is not substantial in this stage. The variation in droplet size is clearly visible in Figure 2c, as the droplets located at the boundaries begin to increase in size, resulting in an anisotropic morphology.

Heat is released to a greater extent during the intermediate and late stages of phase separation, where the difference in concentration between the two phases increases. This effect was comprehensively investigated in our previous studies, and it was concluded that the heat generation increased the solution temperature and could be regarded as a shallow quench effect to some extent during phase separation [8,9]. However, the results provided in this paper reveal that when we consider the temperature- and concentration-dependent density, heat capacity, and heat conductivity in our model development, the rate of heat transfer increases as a result of an increase in the heat diffusivity, which in turn impacts the amount of heat generation due to the increased rate of heat transfer.

In order to gain a more realistic picture of the variations in temperature during phase separation, we need to consider all the factors that might influence heat transfer and, as a result, structure formation. The increase in the heat transfer rate as a result of an increase in heat diffusivity, which influences the phase separation rate and the membrane morphology, outweighs the effect of the enthalpy of the demixing. This is reflected in the concentration and temperature profiles, where more phase separation with smaller droplets is formed as a result of a higher heat transfer rate in comparison to employing a constant heat diffusivity value.

The dimensionless concentration profile and pattern shown in Figure 2d at t* = 8.03 × 10^−4^ demonstrate that on the four sides of the membrane where the quench was applied, the phase separation has entered its final stage and the droplets that were previously formed have merged to reduce the interfacial area due to coarsening. In the interior parts of the sample, the phase separation is still in the intermediate stage. The increase in temperature, as can be seen in the temperature profile in Figure 2d, illustrates the increase in heat generated during phase separation. Terminating the phase separation at any stage would result in a membrane with an anisotropic morphology and desired pore size due to the difference in the size of droplets owing to different rates of heat transfer and phase separation at various parts of the membrane.

If phase separation was allowed to proceed for a considerable time, the concentration and temperature profiles and patterns presented in Figure 2e would result. The droplets that were formed have merged due to coarsening, and the morphology bears resemblance to an interconnected structure. The temperature has almost reached the final quench temperature in the membrane, and the two phases are separated. Comparing the dimensionless concentration and temperature profiles and patterns in this section with the results provided in our previous study reveals that employing the temperature and concentration dependence of the physical properties of the system in phase separation leads to more phase separation being accomplished compared to the simulation results provided in our previous papers [8,9]. This is due to the increased heat transfer rate during phase separation, which is reflected in the temperature and concentration variations in density, heat conductivity, and heat capacity. The variations in each physical property will be discussed in the following paragraphs.

**Figure 3:**
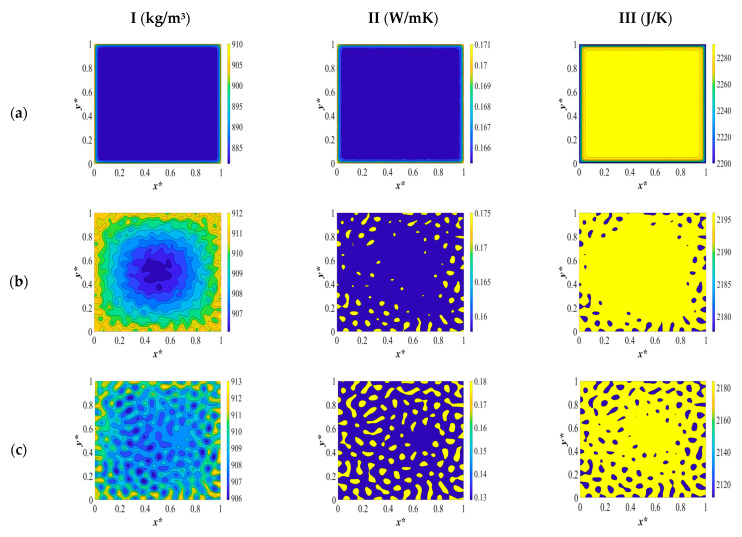

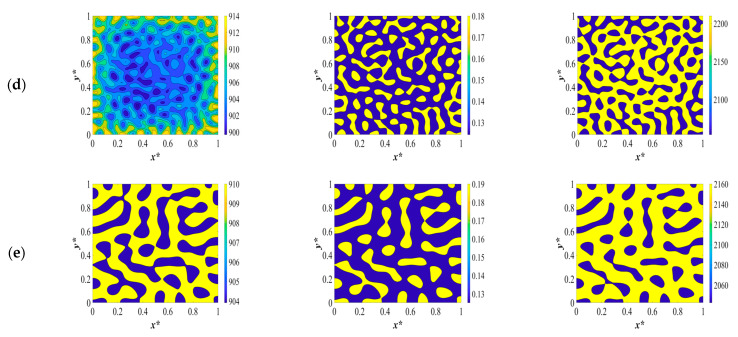
The variations in density (column I), heat conductivity (column II), and heat capacity (column III) during phase separation at the following times: (**a**) t* = 1.03 × 10^−6^, (**b**) t* = 3.03 × 10^−5^, (**c**) t* = 2.03 × 10^−4^, (**d**) t* = 8.03 × 10^−4^, and (**e**) t* = 8.03 × 10^−2^.

The two-dimensional patterns for density (column I), heat conductivity (column II), and heat capacity (column III), plotted at the same time scale as Figure 2 during different stages of phase separation, are provided in Figure 3. The evolution of each physical property is investigated separately during the course of phase separation. Heat transport in a system is governed by the motion of free particles which try to restore thermodynamic equilibrium in the system subjected to a temperature gradient. This phenomenon is discussed in this section in detail.

The dimensionless density pattern is presented in Figure 3I. Initially, the polymer solution at a high temperature has a low density due to the fact that density and temperature have an inverse relationship. When a quench is applied from all sides of the sample at the same time, the density starts to increase. This implies that the phase-separated sample becomes denser than the initial solution, as can be observed in Figure 3(Ia).

As the temperature decreases from the boundaries and develops inside the medium, as presented in Figure 3(Ib), the density increases further. This increase is attributed to the increase in the concentration of the separated phases and the inverse relationship between temperature and density. In other words, due to the uphill diffusion, the molecules of each phase diffuse toward the higher concentrations, which causes the two phases to become denser as the phase separation proceeds. The concentration fluctuations increase gradually during phase separation, which increases density. The off-critical quench with the initial dimensionless solvent volume fraction of c* = 0.85 leads to a droplet-type morphology formation where the majority phase is the polymer-rich phase, and the droplets constitute the solvent-rich phase.

The extent of the increase in density in the sides of the membrane that are exposed to quench is less than it is in the interior parts. This is due to the fact that the sides of the sample are in contact with a cooling medium during the entire phase separation, while in the interior parts, the temperature decreases gradually as the concentrations of each separated phase increase. The temperature continues to decrease in the interior parts, which leads to an increase in density. In other words, there has been no significant change in the concentration to influence the density in the boundaries yet, and the temperature in the boundaries has already reached the quench temperature.

The density pattern corresponding to Figure 2c is presented in Figure 3(Ic). The droplets have started to form on all sides, and the density has started to increase at the boundaries. However, inside the membrane, heat starts to be released as a result of the enthalpy of demixing. This leads to a decrease in density by increasing the temperature of the solution. However, at the boundaries, as a result of being in contact with the cooling medium and the phase separation, which is in progress, the density keeps increasing. The high viscosity of the polymer solution does not impact the temperature at the boundaries due to the enthalpy of demixing. The difference in the concentrations influences the density in each phase.

The amount of heat generation keeps increasing, as can be observed by comparing the dimensionless temperature profiles in Figure 2c,d. This increase in temperature leads to a decrease in density inside the membrane, while at the boundaries of the membrane, density keeps increasing as a result of the high viscosity of the solution and being in contact with a cooling medium. This is a very important phenomenon to take into consideration while investigating the membrane morphology formation during the TIPS process because each of the physical properties is influenced by the temperature and concentration variations during phase separation, which in turn influence the membrane morphology. A deep understanding of the interaction between these parameters leads us to produce membranes with the necessary specifications. In this study, we realize through investigating the influence of the physical parameters on phase separation that the dynamics and thermodynamics of the phase separation process cannot be investigated unless we evaluate all the influential parameters and aspects of this process.

At very late times during phase separation, which corresponds to the dimensionless concentration and temperature profiles in Figure 2e, the two phases are completely separated, and the droplets that were formed during the early and intermediate stages have merged to reduce the interfacial free energy. There is no change in the concentration or temperature, and, as a result, the density remains constant. The density of both phases has increased compared to the initial polymer solution, and, as previously mentioned, temperature and concentration variations together led to an increase in density, and the polymer-rich phase is denser compared with the solvent-rich phase.

The dimensionless heat conductivity patterns during different stages of phase separation are illustrated in Figure 3II. The initial polymer solution at the elevated temperature has a low thermal conductivity. The instant the system is quenched, heat conductivity starts to increase at the boundaries where the quench is applied. This is due to the fact that the thermal conductivity is basically dependent on the motion of free electrons, lattice vibrations, and molecular vibrations. The main mechanism of thermal conduction in liquids is atomic or molecular diffusion. When the temperature decreases, the mean-free path of the molecules increases, which leads to an increase in the thermal conductivity on the quench sides. It is of utmost importance to investigate the variation in the thermal conductivity of the anisotropic materials’ formation. The design and production of polymer materials are constrained by a lack of understanding of heat transfer mechanisms and the significance of thermal conductivity during phase separation. The dependency of thermal conductivity on temperature and concentration during the phase separation process is a critical factor that needs to be taken into account. In liquids, thermal conduction is caused by atomic or molecular diffusion. Since the molecules are close together, there is an intensified interaction when they collide in the thermal conduction mechanism of liquids. Thermal energy is transferred from one molecule or atom to another through thermal conduction. As a result, the heat transfer is affected by the polymer solution’s transport characteristics, which in turn affect the materials’ physical characteristics. Because thermal energy moves more efficiently through polymer chains, the orientation of the chain segments also has a significant impact on the materials’ ability to transmit heat, but this concept is beyond the scope of this study [55,56].

The thermal conductivity of the polymer-lean phase increases during phase separation, and the thermal conductivity of the polymer-rich phase decreases in comparison with the thermal conductivity of the initial polymer solution. The conductivity of the continuous phase decreases, which reduces the total thermal conductivity because the highly conductive solvent-rich droplets will be separated from each other. In amorphous polymers, decreasing the temperature above the glass transition temperature results in an increase in the thermal conductivity, which is reflected in the time evolution of the thermal conductivity patterns presented in Figure 3II.

When phase separation progresses to the time equal to t* = 3.03 × 10^−5^, which corresponds to the dimensionless concentration and temperature profiles and patterns represented in Figure 2b, the droplets of the solvent that are initially formed are more thermally conductive than the polymer solution and the polymer-rich phase. Hence, the thermal conductivity of the solvent-rich droplets increases, and the thermal conductivity inside the membrane also increases due to the increase in the mean-free path of the molecules as a result of the decrease in temperature, which is in accordance with the expected pattern in amorphous polymers.

At time t* = 2.04 × 10^−5^, which corresponds to the dimensionless concentration and temperature profiles depicted in Figure 2c, the thermal conductivity of the solvent-rich droplets continues to increase while the thermal conductivity of the polymer-rich phase decreases. This is due to the fact that the heat that is generated leads to a decrease in the thermal conductivity of the polymer solution. While on the sides of the membrane, the thermal conductivity keeps increasing as a result of the formation of thermally conductive droplets, which merge and increase the conductivity, and are also in contact with the cooling membrane during phase separation. The heat generation due to the enthalpy of demixing influences the thermal conductivity of the polymer solution in a similar way to the density.

The difference between the density and thermal conductivity variations during phase separation is that the polymer-rich membrane that is eventually formed has more density, while the solvent-rich droplets have more heat conductivity. At the late stages of phase separation, where the two phases are completely separated, the thermal conductivity of the solvent-rich droplets, which are now connected due to coarsening, increases while the thermal conductivity of the polymer-rich membrane decreases, making the membrane less thermally conductive.

The third pattern depicted in Figure 3III is the change in heat capacity during the thermally induced phase separation process. The initial polymer solution at a high temperature has a high heat capacity. When the system is quenched by the boundaries, the heat capacity starts to decrease. This is due to the fact that as the temperature decreases, the molecules’ rotations and vibrations decrease, which causes a reduction in the heat capacity. Heat capacity is the slope of the internal energy (enthalpy) with respect to temperature. The internal energy is the energy of molecules, which is the energy due to the rotational and vibrational energy of the molecules. A decrease in temperature leads to a decrease in rotational and vibrational energy. Hence, the sides of the membrane where the quench is applied have a lower heat capacity in comparison to the interior parts. The internal energy decreases due to quenching and increases due to the heat released. In macromolecules, the description of the heat capacity is more complicated. Besides, vibrational motions, large-amplitude rotations, intermolecular rotations, and transitional motions have to be taken into account to investigate the change in heat capacity of the polymer solution.

The heat capacity continues to decrease in the interior parts and in the sides of the membranes at time t* = 3.03 × 10^−5^ which is presented in Figure 3(IIb). The heat capacity of the solvent-rich droplets that are formed during phase separation is lower than the heat capacity of the initial polymer solution. This is also true for the polymer-rich membrane that ultimately forms. The same phenomenon occurs when there is a heat release due to the enthalpy of demixing during phase separation between the times t* = 2.03 × 10^−4^ and t* = 8.03 × 10^−4^ as depicted in the concentration and temperature profiles in Figure 2c,d and the corresponding patterns in Figure 3(IIc,d). As the temperature increases, the heat capacity also increases. At the very late stage of phase separation, the two phases are completely separated, and the heat capacity has decreased in the solvent-rich droplets, which have now merged and formed bigger droplets, as well as in the polymer-rich phase.

**Figure 4:**
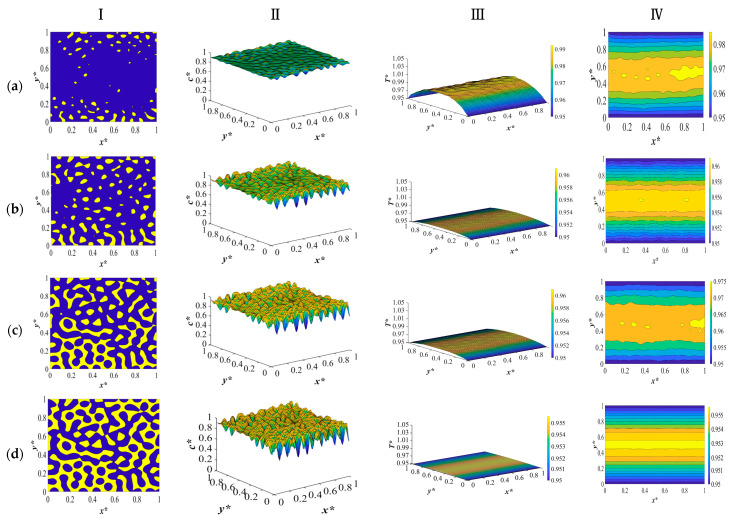
The dimensionless concentration (column I and II) and temperature profiles and patterns (column III and IV) for an off-critical quench with c* = 0.909 and T* = 1.05 and T*_q_ = 0.95 at the following times (**a**) t* = 7 × 10^−4^, (**b**) t* = 1 × 10^−3^, (**c**) t* = 6 × 10^−3^, and (**d**) t* = 9 × 10^−3^.

The results for the second case in Table 3 are provided and discussed in this section. The dimensionless concentration and temperature profiles and patterns for the critical quench of the solution with the initial solvent volume fraction of c* = 0.909 and the initial solution temperature of T* = 1.05 quenched to c* = 0.95 into the two-phase region of the phase diagram are provided in Figure 4. The membrane is quenched from two opposite sides to the same temperature while keeping the other two sides insulated, and the morphology evolution during the thermally induced phase separation process and the variations in the physical properties are investigated. The anisotropic morphology is formed as a result of the difference in the quench rate between the two sides of the membrane. Deep quench leads to the production of relatively smaller droplets, and phase separation is accomplished in a shorter time, which is attributed to the deep quench and the increase in heat diffusivity during phase separation, which will be discussed further.

The results provided in this section are compared with our previous study [8] in terms of the time needed for phase separation and the rate of heat transfer. The comparative analysis shows that the current model exhibits a reduced phase separation time. This is due to the increased rate of heat transfer as a result of the increase in heat diffusivity. A comparison of the results between the models also unveils distinctions in the morphological evolution that occurs during phase separation. Particularly, the variation in the droplet size and the reduction of the demixing heat’s influence during the phase separation process emerge as further dissimilarities between the two models. Notably, there was no substantial variance observed in the computational time between the two models. Consequently, the all-encompassing model introduced in this study more accurately captures the intricacies of phase separation dynamics and thermodynamic behaviors.

The results provided in this section put forward a comparison between the model development employing the physical properties provided in Figure 4 and our previous study with constant heat diffusivity. The critical quench is applied to the system where the initial solution temperature is T* = 1.05 and the quench temperature in the two opposite sides is T* = 0.95. The detailed discussion on the morphology formation is provided in our previous paper for an off-critical quench and a critical quench with the same quench temperatures and boundary conditions [9].

Similar to the results provided in Figure 2, the interaction between the physical properties leads to an increase in the heat diffusivity through increasing heat conductivity and decreasing heat capacity. The rise in these two physical characteristics surpasses the growth in density, ultimately resulting in an elevation of heat diffusivity. This increase in the rate of heat transfer leads to phase separation being accomplished in a shorter time and relatively smaller droplet formation. The results provided in Figure 4 for different periods during phase separation show that when quench is applied from the two opposite sides to the same temperature, the quench rate is the same on both sides. The morphology starts to develop at the boundaries and progresses inside the membrane. Since the rate of heat transfer is higher due to the increase in heat diffusivity, the amount of heat generated is also high.

**Figure 5:**
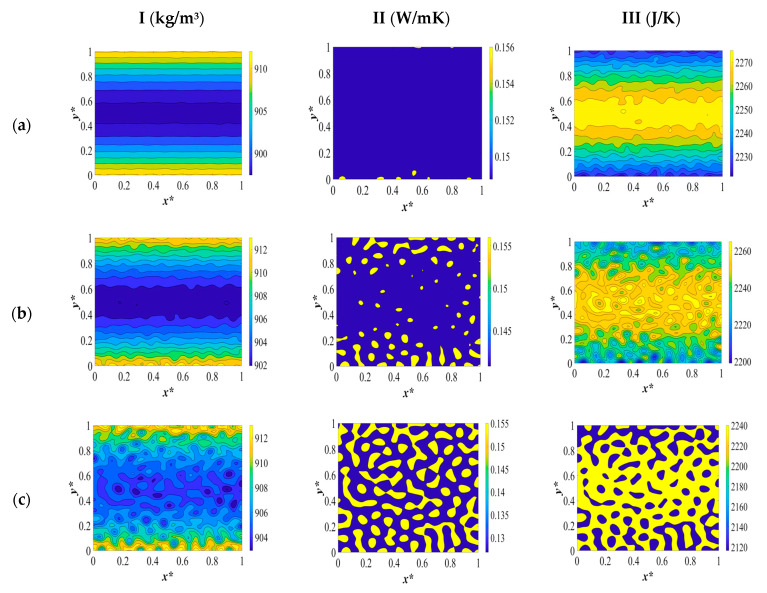

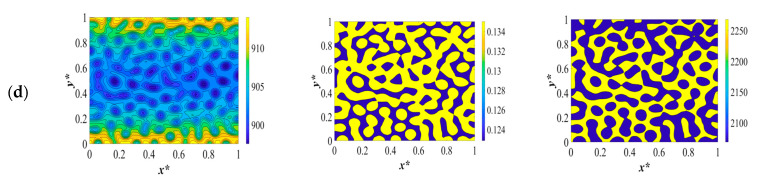
The variations in density (column **I**), heat conductivity (column **II**), and heat capacity (column **III**) during phase separation at the following times*:* (**a**) t* = 7 × 10^−4^, (**b**) t* = 1 × 10^−3^, (**c**) t* = 6 × 10^−3^, and (**d**) t* = 9 × 10^−3^.

Figure 5Ⅰ–Ⅲ represent the density, heat conductivity, and heat capacity patterns during the TIPS process, respectively. The first column on the left side represents the change in density. As can be seen, as the quench is applied, the density starts to increase on both sides. As discussed before, density and temperature have an inverse relationship, and a decrease in temperature leads to an increase in density. As phase separation proceeds inside the membrane, the density increases on both sides and develops inside the membrane. In addition, during phase separation, due to uphill diffusion, the concentration of polymer increases in the polymer-rich phase, while the concentration of solvent increases in the solvent-rich phase., which increases the density of each phase in comparison to the initial homogenous polymer solution.

As presented in the dimensionless density pattern in Figure 5(Ia), density increases on the two sides in the *y*-direction and continues to increase inside the membrane as time progresses. Figure 5(Ib), shows that the density at the boundaries where the quench is applied has not changed yet because there has been no significant change in concentration yet. Nonetheless, inside the membrane, the density keeps increasing. The density increases both inside the membrane and at the boundaries because the phase separation has started and the difference in concentration between the two phases has increased, as illustrated in Figure 5(Ic,d). However, a decrease in the density in the interior parts of the membrane is detected in Figure 5(Ic). This is because the amount of heat generation increases, which leads to a decrease in density.

The middle column illustrates the two-dimensional heat conductivity pattern during phase separation. As discussed before, the heat conductivity of the initial polymer solution is low. As the temperature decreases through the boundaries, heat conductivity starts to increase. This is because the molecular vibrations decrease as the temperature decreases, which in turn increases the mean free path of the molecules. As a result, heat conductivity increases. Heat conductivity keeps increasing at the boundaries and inside the membrane as phase separation progresses. Nonetheless, as presented in Figure 5(IIc), heat conductivity starts to decrease inside the membrane. The reason is the heat generation due to the enthalpy of demixing. However, the thermal conductivity on the quench sides continues to increase. There are two reasons for it. First, as phase separation proceeds, the difference in concentration between the two phases also increases, which leads to an increase in thermal conductivity. Next, due to the high viscosity of the polymer solution, the heat that is generated inside the membrane influences the interior parts more than the quench side because the sides are in contact with the quenching medium during the phase separation.

Thermal conductivity continues to increase from the boundaries where the two phases start to separate, as presented in Figure 5(IIc). The solvent-rich region has higher thermal conductivity than the polymer-rich region. However, the difference in this system with the previous results is because previously we had off-critical quench and a polymer-rich membrane with solvent-rich droplets was formed. However, in this case where we apply quench from two opposite sides, we have a critical quench, which leads to an interconnected-type morphology. Hence, the interconnected morphology represents the solvent-rich region, which has a higher thermal conductivity, and the polymer-rich region shows a lower thermal conductivity. The change in thermal conductivity and, in general, all physical properties should be investigated separately for each quench composition, since the change in physical properties due to different types of membrane morphologies can influence the properties of the membrane.

Column III in Figure 5 represents the variation in the heat capacity during the course of phase separation. Initially, the heat capacity of the polymer solution is high because the temperature of the solution is high. As soon as the quench is administered to the membrane, in the *y* direction, heat capacity starts to decrease because of a decrease in the rotational and vibrational motions of the molecules. This trend continues as phase separation develops in the interior parts until the amount of heat generation increases, as presented in the dimensionless concentration and temperature profiles and patterns in Figure 4d. In this stage, heat capacity increases inside the system in the solvent-rich region. The decrease in the heat capacity is due to the two sides that are in contact with a cooling medium. As phase separation progresses and the interconnected morphology is formed, the heat capacity in the solvent-rich region is lower than that in the polymer-rich region. For amorphous polymers, the change in heat capacity is related to temperature; as temperature decreases, the heat capacity also decreases.

The last set of results reflect the third case provided in Table 3 concerning the variations in concentration and temperature when two opposite sides are quenched to two different temperatures of T* = 0.95 on one side and T* = 0.99 in the opposite side on the *y*-direction with the initial solvent volume fraction of c* = 0.909. A critical quench is applied again in order to compare the morphology formation when we employ the temperature and composition dependence of physical properties.

**Figure 6:**
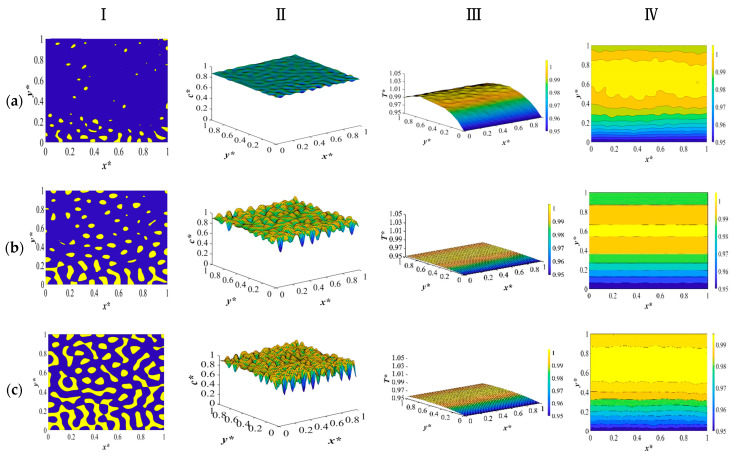
The dimensionless concentration (column I and II) and temperature profiles and patterns (column III and IV) for an off-critical quench with c* = 0.909 and T* = 1.05 and T*_q_ = 0.95 at the following times (**a**) t* = 7 × 10^−4^, (**b**) t* = 1 × 10^−3^, and (**c**) t* = 9 × 10^−3^.

The dimensionless concentration and temperature profiles and patterns are provided in Figure 6. Initially, the solution temperature is high, and the quench is applied at the same time to two opposite sides, as shown in Figure 6a. The side that is quenched to T* = 0.95 shows more phase separation than the side that is quenched to T* = 0.99. This is due to the deep quench effect, which increases the rate of heat transfer and phase separation. As phase separation develops inside the membrane, smaller droplets form on the deep quench side and larger droplets on the shallow quench side. Including the temperature- and concentration-dependent density, heat capacity, and heat conductivity leads to a higher heat transfer rate through increasing the heat diffusivity during phase separation. This leads to the phase separation being completed in a shorter time.

At time t* = 1 × 10^−3^, phase separation in the deep quench side has almost reached its final stage, and the droplets that were previously produced start to merge, while in the shallow quench side, phase separation is still in the intermediate stage. Heat is generated inside the membrane as a result of enthalpy of demixing. Heat generation increases as time passes and the heat moves to the deep quench side and induces a shallow quench effect. This phenomenon has been widely investigated in our previous paper [8,9]. However, comparing the results provided here reveals that, at the same time period during phase separation, the heat transfer rate is high owing to higher heat diffusivity. This leads to smaller droplet formation and weakens the influence of the heat of demixing during phase separation. The reason for choosing temperature and concentration dependence of density, heat conductivity, and heat capacity instead of heat diffusivity alone is to capture the variations of each physical property during phase separation and the influence of the enthalpy of demixing on each physical property.

**Figure 7:**
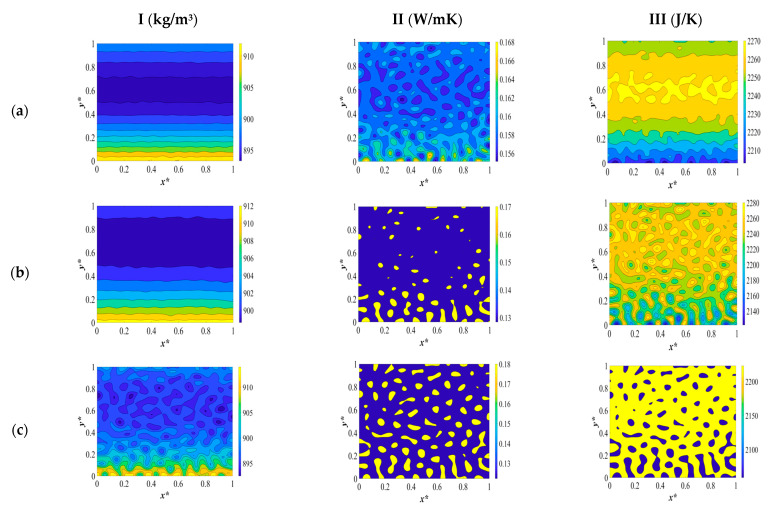
The variations in density (column I), heat conductivity (column II), and heat capacity (column III) during phase separation at the following times (**a**) t* = 7 × 10^−4^, (**b**) t* = 1 × 10^−3^, and (**c**) t* = 9 × 10^−3^.

The dimensionless density, heat conductivity, and heat capacity patterns are provided in picture Figure 7I, Figure 7II and Figure 7III, respectively. As presented in Figure 7(Ia), the side that is quenched deeply shows more change in density than the other side. The initial solution density is low due to the high temperature of the solution and as the quench is applied from the two opposite sides, it starts to increase. The increase in density in the deep quench side is more significant due to the higher cooling rate in the deep quench side. In amorphous polymers, above the glass transition temperature, the density increases when the temperature is decreased. The density increases from the sides and develops inside as the two phases separate. A droplet-type morphology forms as a result of off-critical quench. The solvent-rich droplets that are eventually formed have less density than the polymer-rich membrane and the deep quench side is denser than the shallow quench side as well.

A similar pattern is expected for heat conductivity. Thermal conductivity of the polymer solution is initially low and increases from the boundaries as a result of the quench. For amorphous polymers above the glass transition temperature, when the temperature is reduced, heat conductivity increases until it reaches a maximum at the glass transition temperature and decreases as the temperature is decreased afterward. The thermal conductivity of the solvent-rich droplets is more than the polymer-rich phase and since the droplet-type morphology is formed, the solvent droplets are not connected to each other which decreases the thermal conductivity of the membrane eventually.

On the other hand, the heat capacity of the polymer solution prior the start of phase separation is high and decreases from the boundaries exposed to the quench. As discussed before, the rotational and vibrational movements of molecules decreases with a decrease in temperature, which leads to a decrease in heat capacity. First, the solution has a high specific heat due to high rotational and vibrational movements at a high temperature. Upon quenching the solution, heat capacity starts to decrease until the heat generates in the system which increases the heat capacity. But this effect is not significant, and the heat capacity decreases again. At the end of phase separation, the heat conductivity of the polymer-rich phase is higher than that of the solvent-rich droplets.

In the deep quench side, the increase in density is more significant than the shallow quench side. Likewise, the heat conductivity increases from the deep quench side while in the shallow quench side there is no apparent change in heat conductivity at early times during phase separation. The decrease in heat capacity follows the same pattern i.e., in the deep quench side, the heat capacity starts to decrease. This shows that the droplets of the solvent-rich phase that are being formed have more density, more heat conductivity, and less heat capacity of the initial polymer solution.

Comparing the dimensionless density patterns provide in Figure 5 and Figure 7 reveals that the amount of increase in density inside the membrane is less in the case where quench is applied to two different temperatures (Figure 7) than the case where the same quench temperature is applied in the opposite sides. The reason for that is the quench rate that influences the change in density. In the shallow quench side, the quench rate is lower than the deep quench side which decreases the rate of heat spread inside the membrane. The increase in density in the boundaries is not significant during the early times because the change in concentration has not been started yet. Density inside the membrane increases as phase separation proceeds, until the heat generation prevails and increases the temperature inside the membrane which leads to a decrease in density. Nonetheless, due to high viscosity of the polymer solution and being in contact with the cooling medium during phase separation, the density change in the boundaries is not significant i.e., the enthalpy of demixing does not influence the density change. This phenomenon is observed in the previous two cases investigated in this paper.

A comparison between the heat conductivities of (a) quench from both sides, (b) quench from two sides to the same temperature and keeping the other sides insulate, and (c) quench from two sides to two different temperatures reveals that higher initial volume fraction of the solvent leads to higher values of the solution thermal conductivity. This is expected because the solvent is more conductive than the polymer, and a higher volume fraction of the solvent leads to a higher initial thermal conductivity of the polymer solution. The thermal conductivity increases more from the deep quench side than from the shallow quench side and continues to increase in the interior parts as the temperature of the membrane decreases and the phase separation progresses. However, on the side that is exposed to a shallow quench, the thermal conductivity is lower than on the side where the solvent droplets are already formed. As phase separation progresses, the solvent-rich phase separates as droplets, which have a higher conductivity than the polymer-rich phase. This can be seen in the dimensionless heat conductivity profile in Figure 7(IId). All the results provided in this paper emphasize the influence of physical properties during phase separation in order to regulate the process and produce materials with desired morphologies.

We compared the outcomes presented with our prior research [9]. Through this comparison, it becomes evident that the current model showcases a decreased phase separation duration, attributed to the heightened heat transfer rate resulting from an increase in heat diffusivity. Moreover, a contrast between the models’ outcomes also unveils apparent dissimilarities in the morphological evolution that develops during the phase separation process. Specifically, variations in droplet size and the reduction of demixing heat’s influence come to the forefront as additional disparities between the two models. It is important to note that no significant discrepancy in computational time was observed between the two models. Consequently, the comprehensive model introduced in this study provides a more precise depiction of the intricate dynamics of the phase separation process.

## 4. Conclusions

In this research, the relationship between the morphology formation of a high-viscosity polymer solution during thermal phase separation and the composition- and temperature-dependent density, heat capacity, and thermal conductivity was investigated. The study combined the Fourier heat transfer equation and the Cahn–Hilliard equation to investigate the effect of these properties on the phase separation process. The study also analyzed the variations of each physical property under different boundary conditions and initial concentrations. The results showed that the physical properties of the polymer solution have a significant impact on the morphology formation during phase separation. Additionally, the role of enthalpy of demixing was found to be crucial, as the heat generated during phase separation leads to non-uniform patterns in each physical property, affecting the formation of morphology. The findings indicate that simply considering phase separation dynamics and thermodynamics without heat transfer would not provide a complete understanding of the process and its impact on morphology formation. Further research is needed to fully comprehend the dynamics of spinodal decomposition, heat transfer, and the influence of each physical property on morphology formation in high-viscosity polymer solutions and blends.

The comparison made between the model developed in this study with the models of our previous papers revealed that the current model demonstrates shorter phase separation time and an enhanced heat transfer rate as a result of increased heat diffusivity. Additionally, a comparison of the models’ outcomes highlights distinctions in the morphological evolution during phase separation, particularly in terms of droplet size variation. Despite these differences, the computational time remains largely consistent between the models. Consequently, the comprehensive model introduced in this study offers a more accurate depiction of the phase separation process including thermal effects.

## Figures and Tables

**Table 1:** The properties and the parameters utilized in the model development.

**The Property**	The Equation Utilized in the Model Development	Eq.	References
The total free energy of a system	$F=\int \left[f\left(c\right)+\kappa {\left(\nabla c\right)}^{2}\right]dV$	(1)	[2,29,31]
The free energy of the homogenous system	$f\left(c\right)=\frac{k_{B}T}{\nu }\left[\frac{c}{N_{1}}lnc+\frac{1-c}{N_{2}}\ln\left(1-c\right)+\chi c(1-c)\right]$	(2)	[32]
Flory’s interaction parameter	$\chi =\frac{V_{cyclohexanol}}{RT}{\left(\delta_{cyclohexanol}-\delta_{polystyrene}\right)}^{2}+0.34$	(3)	[23,33]
The solubility parameters of the solvent and the polymer	${\delta_{polystyrene}}^{2}={\delta_{p}}^{2}+{\delta_{h}}^{2}+{\delta_{d}}^{2}=22.68$ ${\delta_{cyclohexanol}}^{2}={\delta_{p}}^{2}+{\delta_{h}}^{2}+{\delta_{d}}^{2}=22.401$	(4)	[34,35]
The nonlinear Cahn-Hilliard equation	$\frac{\partial c}{\partial t}=\nabla \cdot \left[M\nabla \left[\frac{\partial f}{\partial c}-2\kappa \nabla^{2}c\right]\right]$	(5)	[36,37]
The mobility	$M=\frac{\nu c\left(1-c\right)}{\xi }$	(6)	[1,15,27,30]
The gradient energy coefficient	$\kappa =\frac{RT\chi l^{2}}{6}$	(7)	[36,38,39,40,41]

**Table 2:** The equations utilized to designate the temperature- and composition-dependence of physical properties.

**Property**	Solvent (Cyclohexanol)	Polymer (Polystyrene)
Specific heat capacity (J/kg·K)	$c_{p,s}=-A+BT-CT^{2}+DT^{3}$ ^a^ A=470,B=19 C=47,D=48	$c_{p,p}=A+BT$ ^b^ A=1049.2,B=2.236
Density (kg/m^3^)	$\rho_{s}=\frac{A}{B^{(1+{\left(1-\frac{T}{C)}\right)}^{D}}}$ ^c^ A=82.43 B=0.26546 C=650 D=0.2848	$\rho_{p}=A-BT-CT^{2}$ ^d^ A=1.067 $B=5.02\times 10^{-4}$l $C=0.135*10^{-6}$
Heat conductivity (W/m·K)	$k_{s}=A-BT$ ^e^ A=0.2092 $B=2.5*10^{-4}$	$k_{p}=k\left(T_{g}\right)*\left(1.2-\frac{0.2T}{T_{g}}\right)$ ^f,g^

Temperature is in Kelvin. ^a^ Data from Ref. [49], ^b^ Data from Ref. [50], ^c^ Data from Ref. [51], ^d^ Data from Ref. [52], ^e^ Data from Ref. [51], ^f^ Data from Ref. [52], ^g^ *T_g_* is the glass transition temperature.

**Table 3:** The initial solvent volume fractions and the initial and quench temperature selected in this study.

Case	Initial Solvent Volume Fraction	Initial Solution Temperature	Quench Temperature
1	c* = 0.85	T* = 1.03	T*_q_ = 0.97
2	c* = 0.909	T* = 1.05	T*_q__1_ = 0.95 T*_q__2_ = 0.95
3	c* = 0.909	T* = 1.05	T*_q__1_ = 0.95 T*_q__2_ = 0.99

## Data Availability

The data presented in this study are available on request from the corresponding author.
